# Graphite-Based Geothermometry on Almahata Sitta Ureilitic Meteorites

**DOI:** 10.3390/min10111005

**Published:** 2020-11-12

**Authors:** Anna Barbaro, M. Chiara Domeneghetti, Cyrena A. Goodrich, Moreno Meneghetti, Lucio Litti, Anna Maria Fioretti, Peter Jenniskens, Muawia H. Shaddad, Fabrizio Nestola

**Affiliations:** 1 Department of Earth and Environmental Sciences, University of Pavia, 27100 Pavia, Italy;; 2 Lunar and Planetary Institute, Universities Space Research Association, Houston, TX 77058, USA;; 3 Department of Chemical Sciences, University of Padova, 35131 Padova, Italy;; 4 Institute of Geosciences and Earth Resources, National Research Council, 35131 Padova, Italy;; 5 SETI Institute, Mountain View, CA 94043, USA;; 6 Department of Physics and Astronomy, University of Khartoum, Khartoum 11111, Sudan;; 7 Department of Geosciences, University of Padova, 35131 Padova, Italy;; 8 Geoscience Institute, Goethe-University Frankfurt, 60323 Frankfurt, Germany

**Keywords:** ureilites, meteorites, carbon phases, graphite, graphite geothermometer, shock event

## Abstract

The thermal history of carbon phases, including graphite and diamond, in the ureilite meteorites has implications for the formation, igneous evolution, and impact disruption of their parent body early in the history of the Solar System. Geothermometry data were obtained by micro-Raman spectroscopy on graphite in Almahata Sitta (AhS) ureilites AhS 72, AhS 209b and AhS A135A from the University of Khartoum collection. In these samples, graphite shows G-band peak centers between 1578 and 1585 cm^−1^ and the full width at half maximum values correspond to a crystallization temperature of 1266 °C for graphite for AhS 209b, 1242 °C for AhS 72, and 1332 °C for AhS A135A. Recent work on AhS 72 and AhS 209b has shown graphite associated with nanodiamonds and argued that this assemblage formed due to an impact-event. Our samples show disordered graphite with a crystalline domain size ranging between about 70 and 140 nm. The nanometric grain-size of the recrystallized graphite indicates that it records a shock event and thus argues that the temperatures we obtained are related to such an event, rather than the primary igneous processing of the ureilite parent body.

## 1. Introduction

Almahata Sitta (AhS) is the first meteorite to originate from a known asteroid, 2008 TC_3_. This asteroid was discovered on 6 October 2008 and tracked for ~20 h before it hit Earth in the Nubian Desert, Sudan [1,2]. The AhS meteorites in the University of Khartoum (UoK) collection consist of >~700 cm-sized stones of diverse meteorite types [2–4]. Those studied so far are dominated by ureilites, which are a major group of achondrites, but also include several types of chondrites (enstatite, ordinary, carbonaceous and Rumuruti chondrites are a range of subtypes) [3].

AhS is classified as an “anomalous polymict ureilite” [1]. It is analogous to typical polymict ureilites, which are fragmental breccias dominated by ureilitic clasts, except that it disintegrated in the atmosphere with its clasts landing on Earth as individual stones [4]. The ureilitic clasts in polymict ureilites, including AhS, are essentially identical to main group ureilites, except possibly that a higher fraction of them are highly shocked [5–7].

Ureilites are ultramafic rocks mainly composed of olivine and pyroxene, with minor carbon phases, metals, and sulfides. The most common pyroxene in most ureilites is pigeonite. A few ureilites contain augite and orthopyroxene instead of or in addition to pigeonite [4]. They are interpreted to represent a single original ureilitic parent body (the UPB), which accreted within 1–2 Ma after CAI (Calcium Aluminum Inclusions) formation and shortly thereafter was partially differentiated, experiencing igneous processing at temperatures up to 1200–1300 °C [4]. It was then disrupted by a catastrophic impact at ~5 Ma after CAI, followed by reassembly of daughter bodies from which the known ureilites probably originate [4–8]. Various degrees of shock recorded in ureilite silicates (e.g., [7,9,10]) may result largely from this event, although the reassembled bodies likely experienced subsequent impact events as well, including the recent breakup events that brought fragments of them into Earth-crossing orbits.

The carbon in ureilites occurs dominantly as graphite, in elongated masses along silicate grain boundaries. There is compelling evidence (from very low shock ureilites) that the primary form of graphite in all ureilites was mm sized crystals of well-crystalline graphite [11–13]. Diamonds in ureilites always occur embedded in the graphite masses.

Some recent studies [14,15] have proposed formation of diamonds at static pressures >20 GPa in a large planetary body, similar to the diamonds formed deep within the Earth’s mantle. This would imply that the UPB was a large planetary embryo, the former existence of which is predicted by current planetary formation models [15]. In contrast, Goodrich et al. [16] and Nestola et al. [17] showed that there is no evidence supporting the requirement of long growth times at high static pressures and argued for the formation by shock transformation from originally larger graphite crystals. Understanding the origin of the diamonds critically depends on constraining the thermometric and shock history of the graphite in which they are embedded, which is the subject of this investigation. [17] showed that graphite can be nanometric in size in shocked ureilites.

In order to elucidate the nature of graphite in different ureilitic fragments of AhS, we applied a graphite-based geothermometer (recently applied to chondrites by [18], and to other AhS ureilites by [19]) on these fragments (two of which were studied by [17]). In addition, using published calibrations on the ratio of Raman D-bands and G-bands intensities [20,21], we were able to determine the crystallite size of graphite, which could add crucial information regarding the thermal and crystallization/re-crystallization history of the graphite.

## 2. Materials and Methods

Our study was performed on Almahata Sitta (AhS) samples AhS 72, AhS 209b and AhS A135A, which are three stones from the Almahata Sitta meteorite that fell in the Nubian desert in 2008 [2]. These samples belong to the collection of the University of Khartoum, Sudan.

The petrographic description was carried out on the AhS 209b and AhS 72 polished sections. We obtained backscattered electron images (BSE) of non-carbon-coated sections of AhS 209b, AhS 72 and of the main mass (embedded in epoxy) of AhS A135A. For the AhS A135A sample, the SEM analysis was performed on just a tiny mass embedded in epoxy, as this was the only available sample.

The investigation by SEM on carbon aggregates was conducted at the Astromaterials Research and Exploration Science Division at the Johnson Space Center in Houston, Texas (USA) using the JEOL 5910-LV SEM (JEOL Ltd., Tokyo, Japan) and at the Centro Analisi per la Certificazione (CEASC) at the University of Padova (Italy) using the FEI Quanta 200 (FEL, Brno, Czech Republic), low vacuum SEM. The observations using the JEOL 5910-LV SEM were made at 15 KeV accelerating potential in normal high vacuum mode, despite the lack of carbon coat, in order to allow higher beam currents (and hence greater BSE contrast). Under these conditions charging of silicates was observed, but carbon areas were sufficiently conductive to provide good images. The BSE images of AhS A135A were obtained using the FEI Quanta 200 SEM, using 20 KeV accelerating potential in low vacuum mode. Silicate mineral compositions were determined by electron microprobe analyses (EMPA) at the Johnson Space Center, with techniques and results described in [17].

The polishing and cutting procedure for preparing the samples can induce defects on graphite crystals [22]. As reported by these authors, the polishing procedure could induce an unpredictable increase in the ID/IG ratio [integrated intensity(D-band)/integrated intensity(G-band)], which in this work has been used to determine the crystallite size [20], while it does not affect the Full With at Half Maximum (FWHM) of (G) parameter [18]. For this specific reason, we conducted our Raman spectroscopy on unpolished carbon-bearing samples. This non-destructive approach is crucial for providing reliable estimation of crystallite size by Raman spectroscopy of graphite.

**Figure 1.**
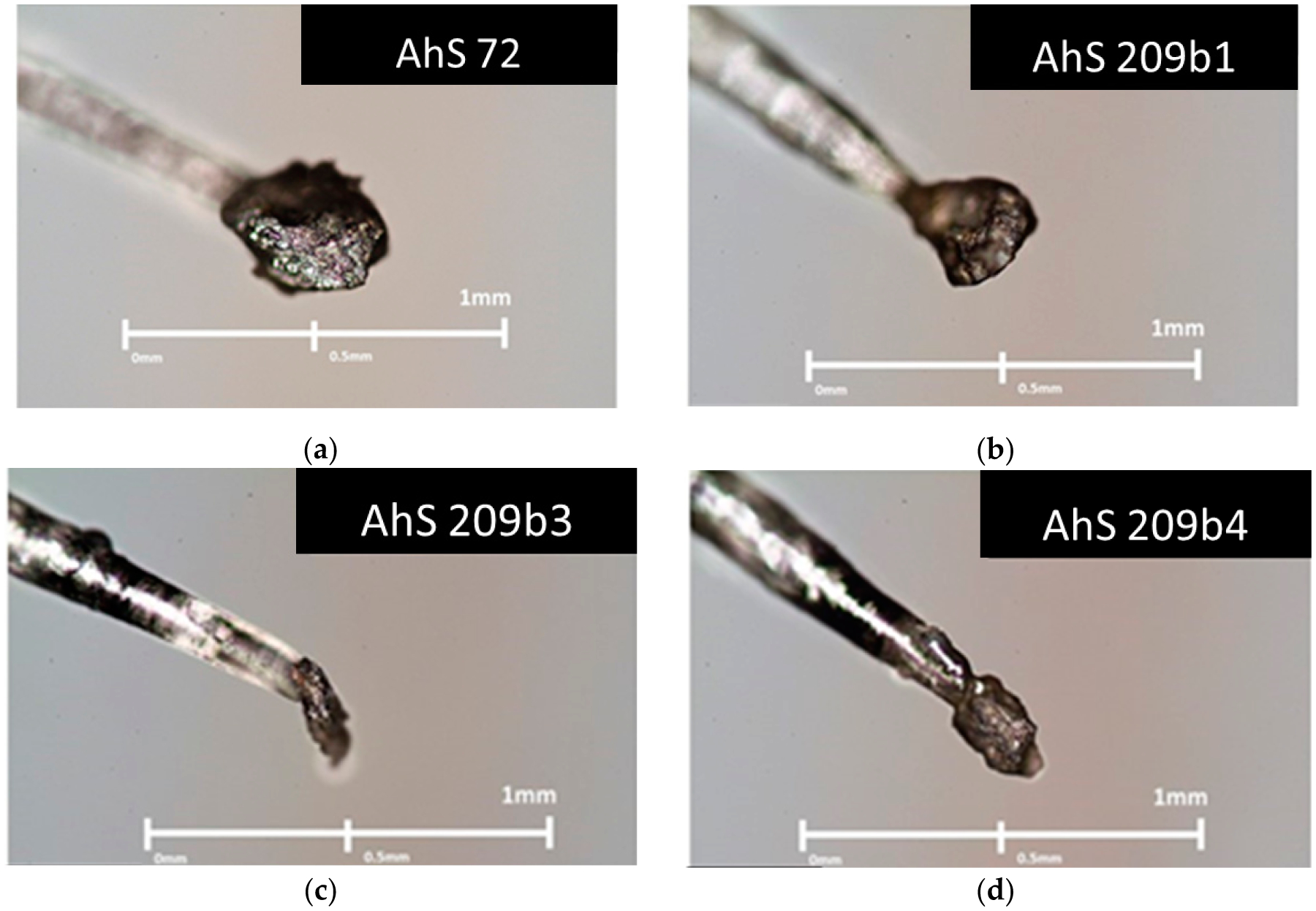

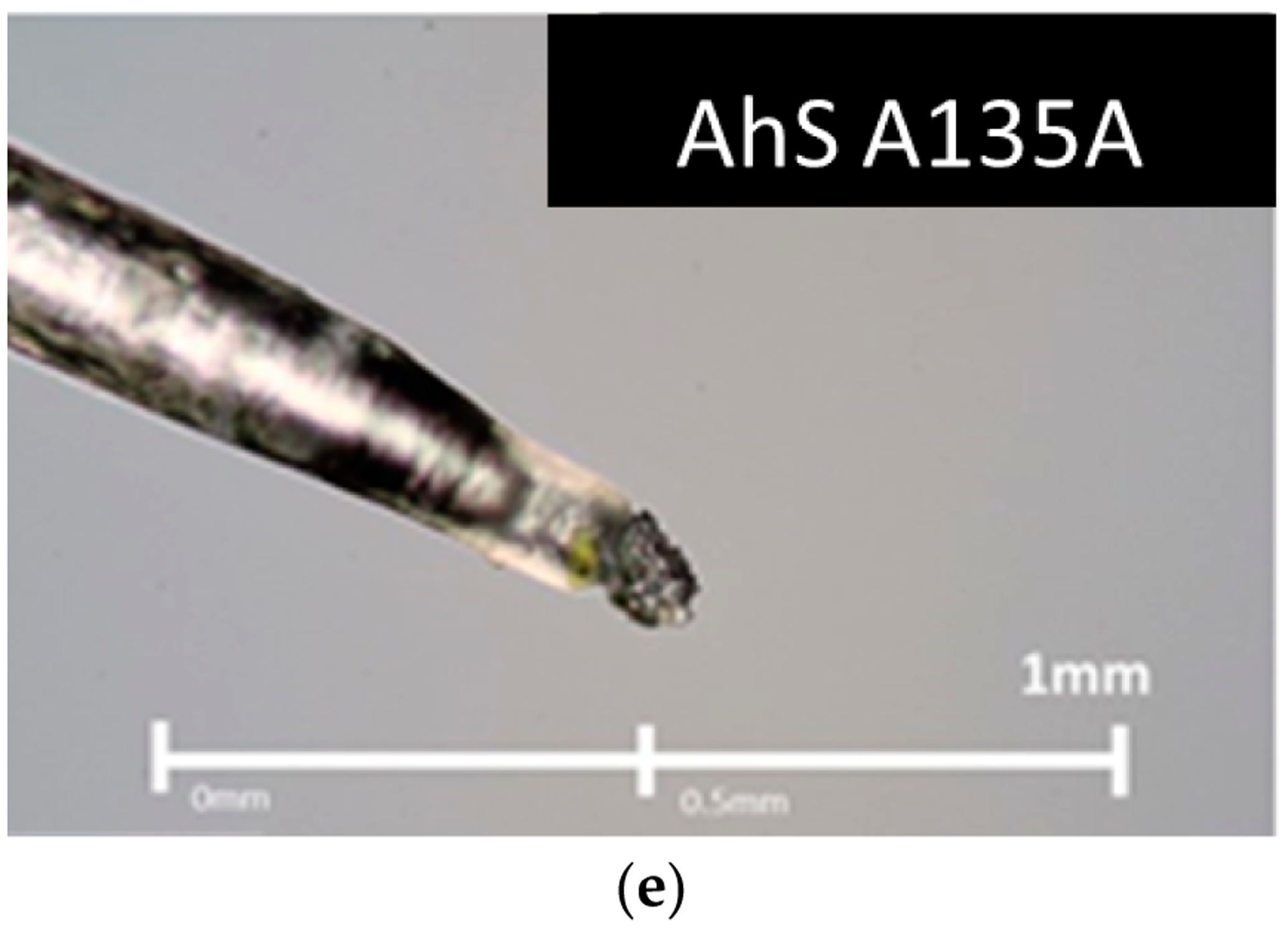
Graphite-bearing fragments glued on top of glass fibers 0.1 mm thick. (**a**) Almahata Sitta (AhS) 72. (**b**) AhS 209b1. (**c**) AhS 209b2. (**d**) AhS 209b4. (**e**) AhS A135A.

Graphite-bearing fragments, with sizes ranging between 0.10 and 0.50 mm, were gently removed from the AhS ureilites and only non-polished volumes of such fragments were glued on top of 0.10 mm thick glass fibers (Figure 1).

Confocal micro-Raman Spectroscopy (MRS) analysis was conducted on the graphite fragments (e.g., Figure 1) using an inVia Renishaw micro-Raman spectrometer installed at the Department of Chemical Sciences of the University of Padova. We used a 514 nm laser excitation with an operating power of 1.3 mW, in order to avoid any graphite damage. A magnification of 50× was used for AhS 209b and AhS 72 samples while for AhS A135A we used a magnification of 100×. The spectral resolution was 1.5 cm^−1^, the laser beam spot on the samples was about 1 μm. For each sample, we used a 30 s integration time with five accumulations for each spectrum. The spectra were always collected very far from the areas in contact with the fiber glass to avoid any Raman signal coming from the glue. A high-quality octahedral gemstone lithospheric diamond was used as a standard material to obtain the instrumental broadening, following the same experimental procedure used in [19] (see section on geothermometry below). Curve fitting of the spectra was carried out using the software OMNIC^™^ for dispersive Raman (Thermo Fisher Scientific, Waltham, WA, USA), adopting Gaussian and Lorentzian curves to obtain the best fit.

## 3. Results

### 3.1. Petrographic Analysis: Characterization of AhS Graphite Phases

**Figure 2.**
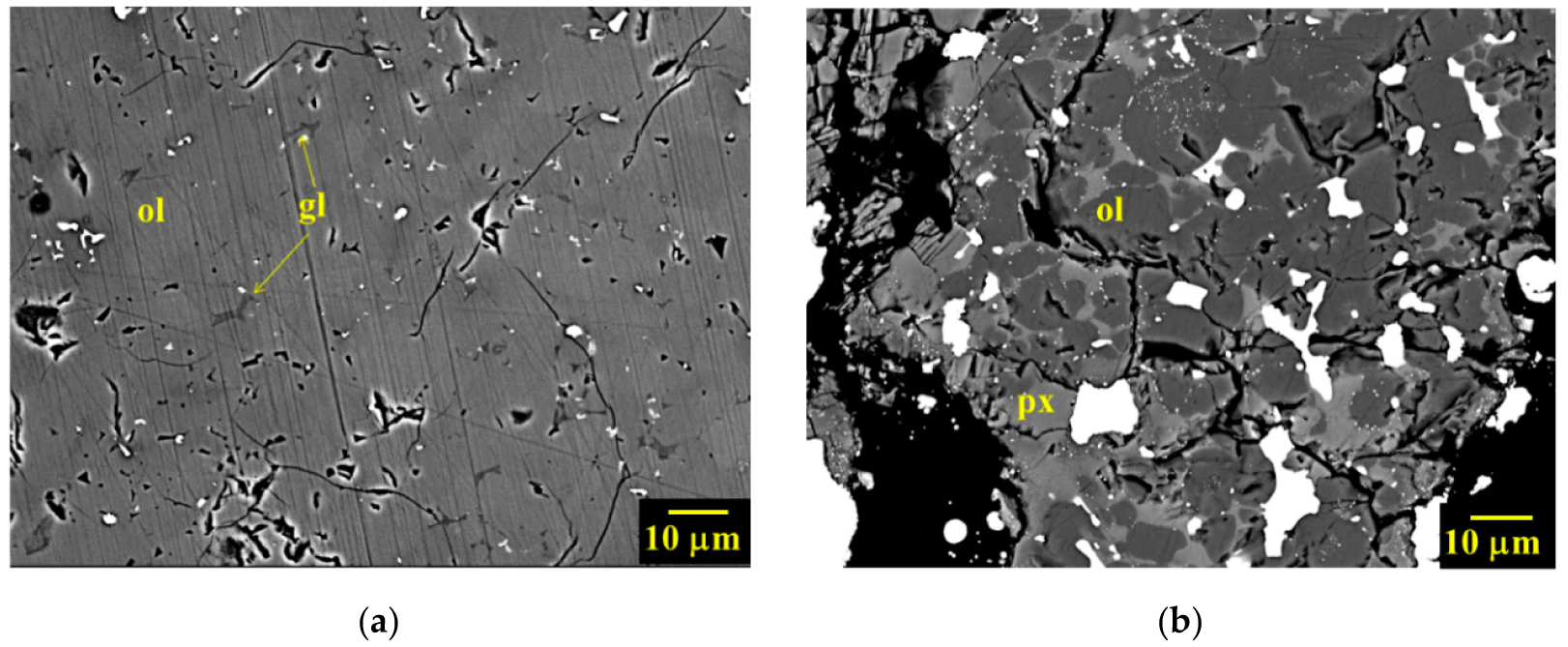

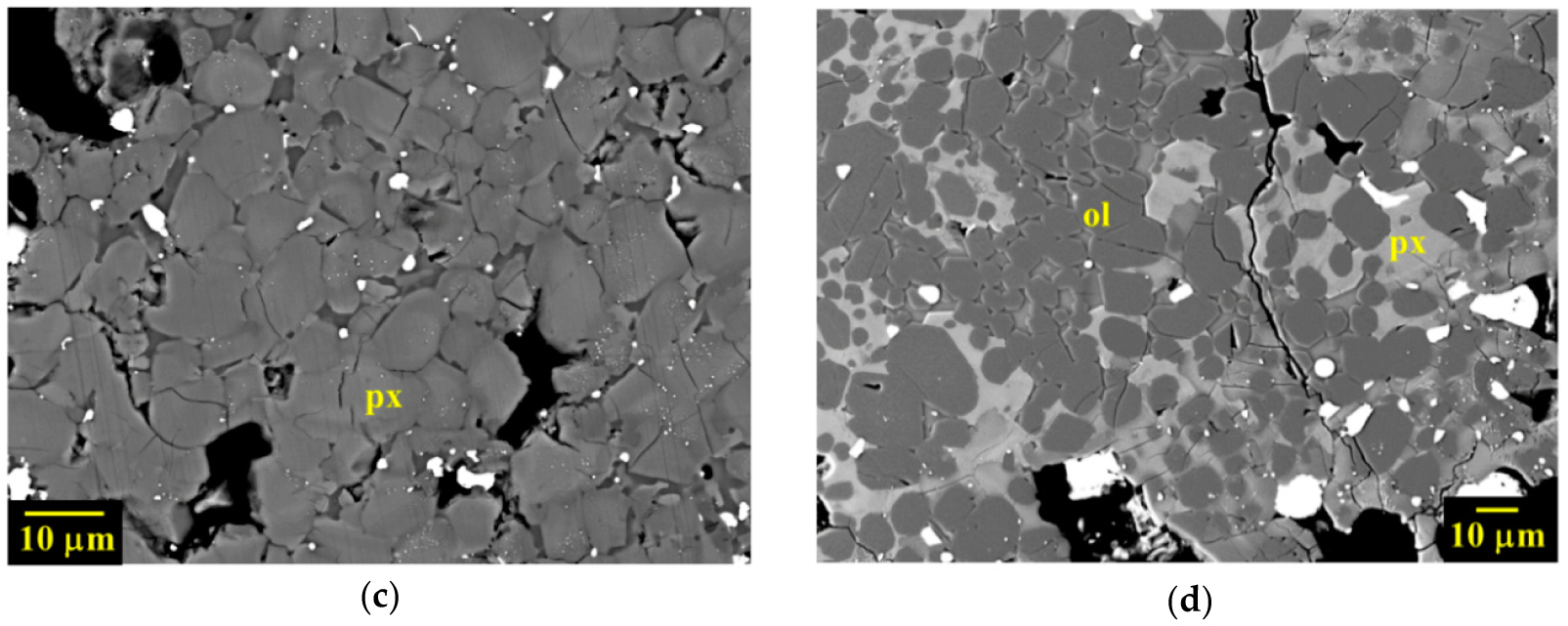
(**a**) Back-scattered electron image (BSE) of AhS #209b, showing the dominant texture of olivine areas (the parallel lines on the surface are polishing scratches). Original olivine (ol) crystals are completely mosaicized to ~5–20 μm sized equigranular tiles, with minor interstitial Si-Al-enriched glass (gl). (**b**) BSE of less common, impact-smelted olivine area in AhS 209b, with ~5–20 μm sized equigranular, rounded, grains of reduced olivine with interstitial pyroxenes (px). Metal and sulfide grains (bright) are common. (**c**) BSE of AhS #209b showing impact-smelted pyroxene, consisting of aggregates of ~5–10 μm sized subhedral grains, with small amounts of interstitial Ca-enriched pyroxenes (px) and Si-Al-enriched glass. Pores and small grains of metal and sulfide (bright) are common. Pyroxenes are reduced relative to inferred primary compositions and show further-reduced outer rims. (**d**) BSE showing dominant lithology in AhS #72, similar to (**b**), of equigranular, rounded, highly reduced olivine with interstitial pyroxene.

Fragments AhS 209b and AhS 72 (Figure 2) are fine-grained, porous ureilites showing various degrees of “impact-smelting” and shock metamorphism as previously described for fine-grained AhS ureilites and a few main group ureilites [23].

**Figure 3.**
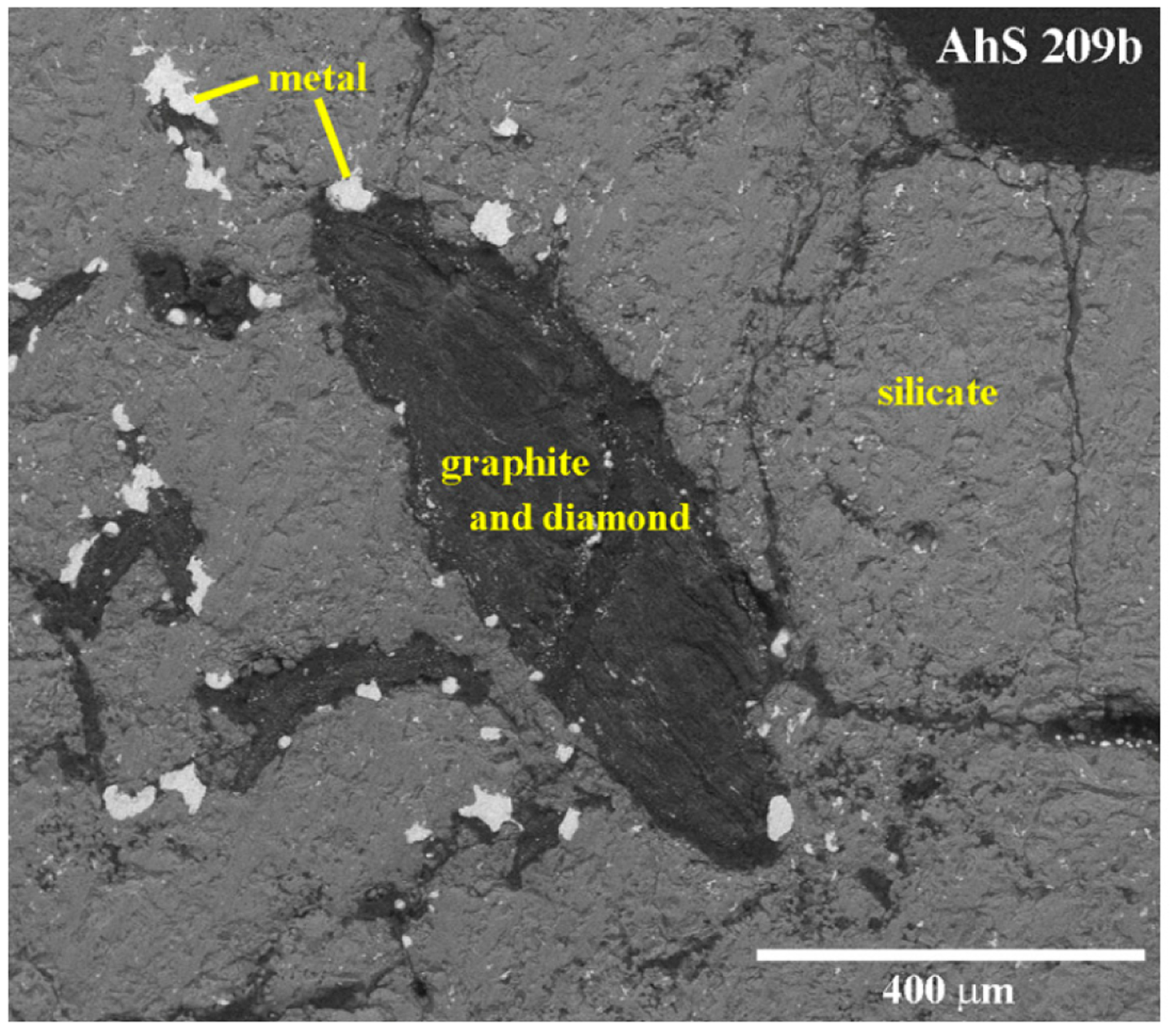
Carbon area in polished sections of AhS 209b. Back-scattered electron image (BSE) of the non-carbon-coated section of AhS 209b collected in low-vacuum mode.

**Figure 4.**
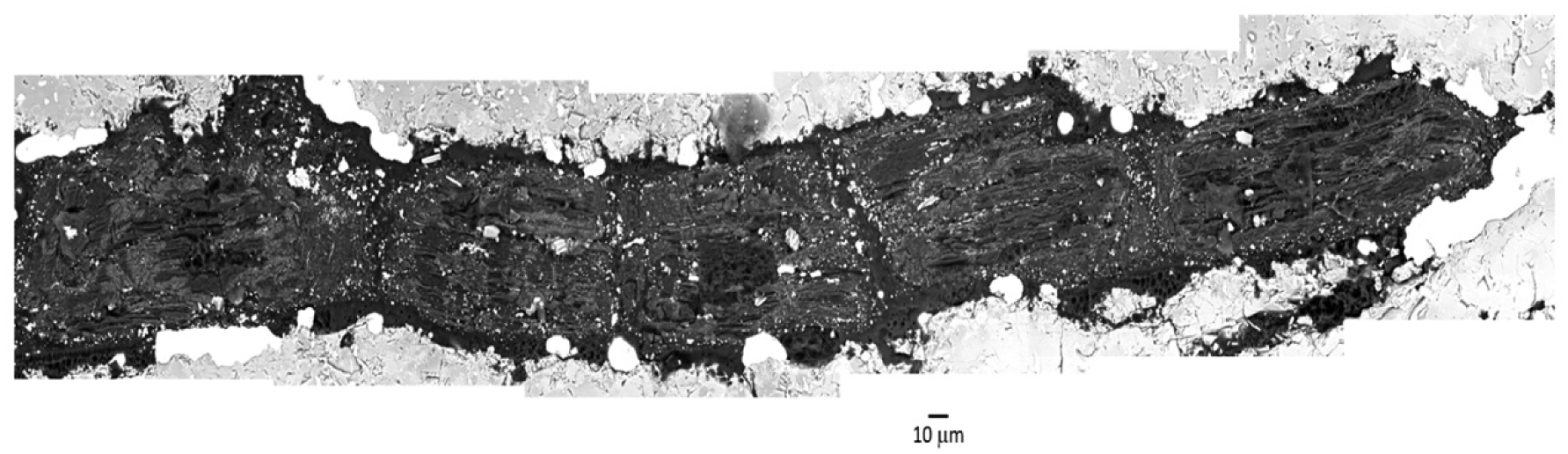
Collage of six BSE images showing blade-shaped carbon area in AhS 209b.

It is evident in Figure 2 that olivine areas in AhS 209b are completely mosaicized. They consist of aggregates of ~5–20 μm sized equigranular tiles (adopting the terminology of [23]) with tiny amounts of interstitial pyroxene and Si-Al-rich glass. The outlines of the original larger (~mm sized) primary silicate grains are defined by cracks, aggregates of carbon phases and metal as seen in Figures 3 and 4.

The olivine largely preserves a typical ureilite olivine core composition of Fo ~79, except in reduction rims near original grain boundaries and/or graphite aggregates. Reduction rim compositions range up to Fo ~93. Pigeonitic pyroxene areas in AhS 209b also show complete mosaicism with extensive in-situ reduction and porosity. They consist of aggregates of ~5–10 μm sized subhedral to anhedral grains, with varying amounts of interstitial Ca-enriched pyroxenes and Si-Al-enriched glass. Pores and small grains of metal and sulfide among the pyroxene grains are common. The pyroxene tiles show reverse zoning. Cores are reduced (core Mg#s up to ~93) relative to inferred primary compositions (~Mg# 81, such as would have been in equilibrium with Fo ~79 olivine) with varying Wo contents (~2–8). Dustings of very fine metal grains occur in some of the cores, indicating multiple episodes of reduction. Pyroxene textures such as these were described by [23] in several main groups and Almahata Sitta ureilites and were attributed to “impact smelting”.

The fragment of AhS 72 that we examined is dominated by olivine and shows a higher degree of shock metamorphism than AhS 209b. Olivine is completely re-crystallized to ~1–20 μm sized equigranular (anhedral to subhedral) grains in a groundmass (of varying proportions relative to the amount of olivine) of pyroxene. The olivine grains are highly reduced (Fo ~99) and nearly free of inclusions, suggesting re-crystallization from a melt (or at least at very high temperatures) under highly reducing conditions [17]. Interstitial pyroxene compositions range from Wo 0.8 to Wo 34 and are also reduced (Mg# 88–99). Pores, masses of graphite, and grains of metal are abundant and generally on a much larger scale (~20–100 s of μm) than the olivine grains. As also reported by [16,17], it is evident from Figures 3 and 4 that in samples AhS 209b and AhS 72, the carbon aggregates typically occur as elongated (blade-shaped), internally layered structures of up to 1 mm in length and 300 μm in width (Figure 2) located along original silicate grain boundaries. The lighter areas contain numerous tiny, bright grains of what appears to be mainly Fe-sulfides, based on the EDS spectra showing peaks for C, Fe, and S. The darker areas appear to be largely free of inclusions and have EDS spectra showing only C.

**Figure 5.**
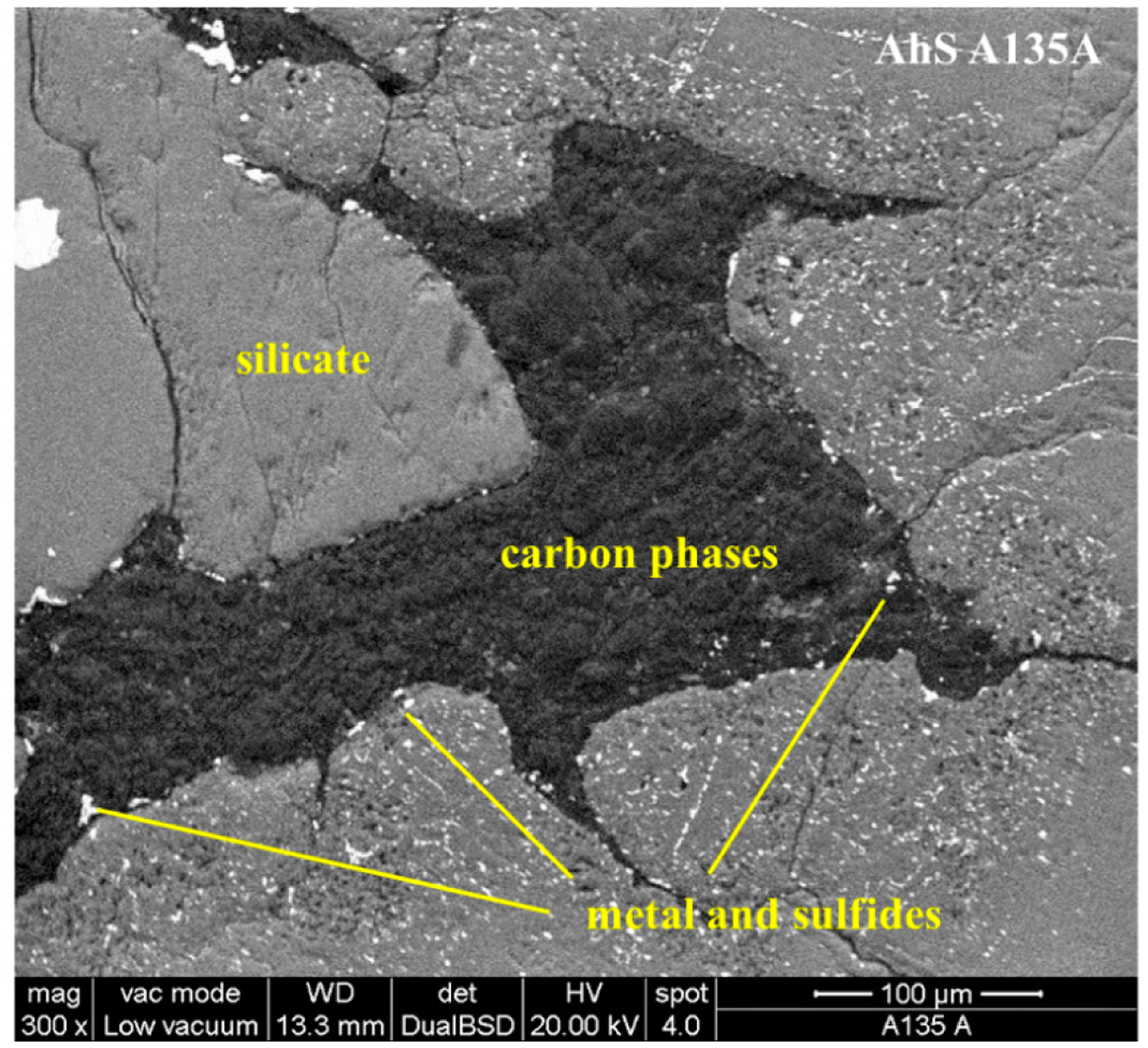
Carbon area in AhS A135A. Back-scattered electron image (BSE) of the non-carbon-coated surface of AhS A135A collected in low-vacuum mode.

AhS A135A is composed of olivine and minor low Ca pyroxene and metal-sulfide blebs. AhS 135A is classified as a typical coarse-grained ureilite with a medium shock level. As in most ureilites, carbon masses occur principally in elongated shapes along silicate grain boundaries. Carbon phases are intermixed with minor Fe and Ni compounds and sulfides (Figure 5).

### 3.2. Micro-Raman Analysis: Characterization of AhS Graphite Phases

**Figure 6.**
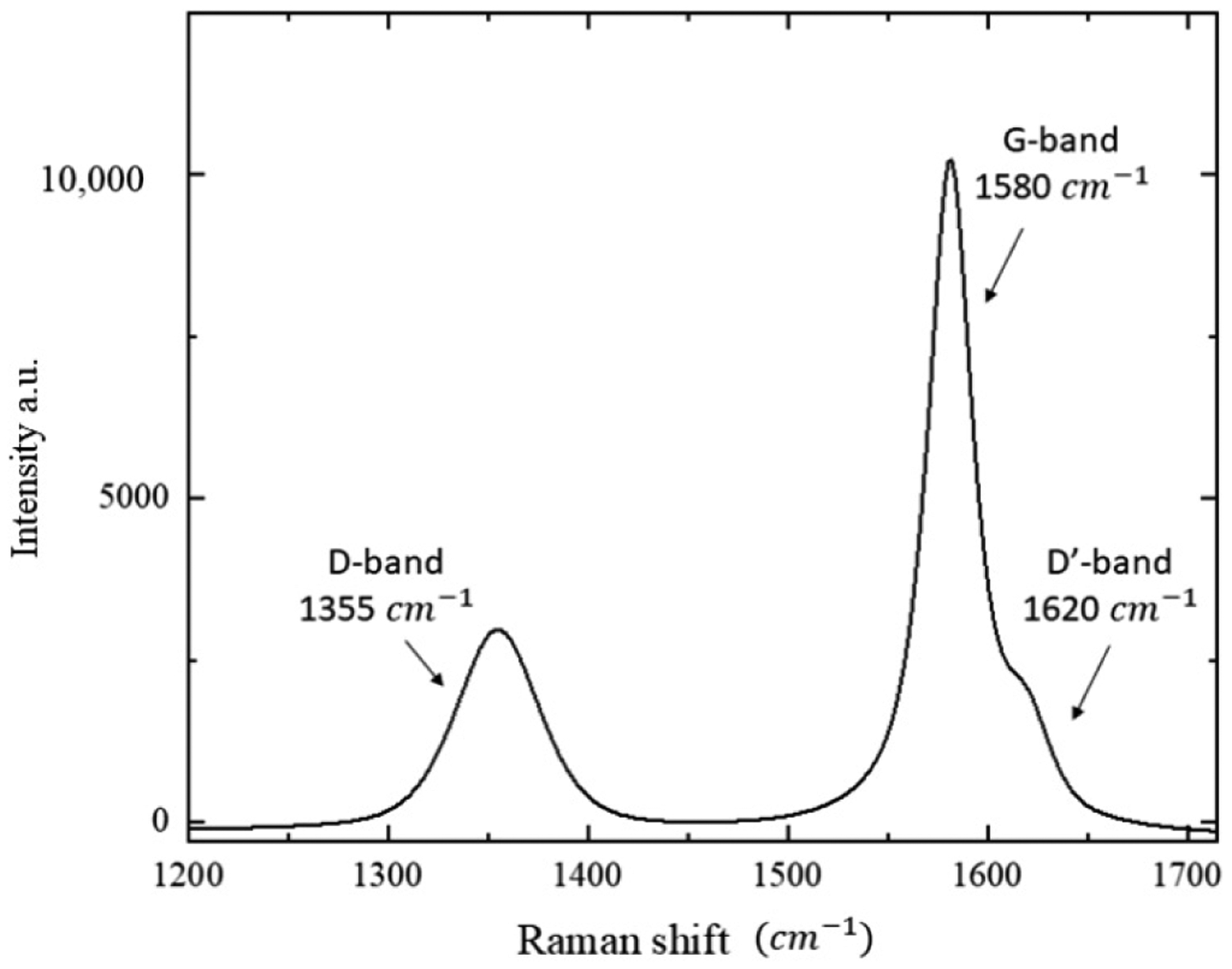
A Raman spectrum of graphite in AhS 209b. The band positions are indicated in the spectrum: G-band at 1580 cm^−1^, D-band at 1355 cm^−1^ and D’-band at 1620 cm^−1^.

Figure 6 shows a typical Raman spectrum of graphite in our AhS samples. The spectra of all samples investigated are practically identical. They show three Raman bands: G- and D-bands together with the D′-band (following the same nomenclature used by [21]). The G-band is at around 1580 cm^−1^, which is the main band of crystalline graphite; the D-band is at around 1355 cm^−1^, which is defect-induced and is the band that refers to the disordered graphite [21]. In almost all samples, the D′-band at around 1620 cm^−1^ is detected as a shoulder of the G-band peaks.

Table 1 shows the I(D)/I(G) ratio (where I = integrated intensity; D = D-band; G = G-band) for all the studied samples, representing the ratio of the integrated D- and G-band intensities. These values range between 0.3 and 0.9.

An important relationship between the ratio of the intensity of D-band and G-band (I(D)/I(G)) and the crystallite size of graphite (*L*_*a*_) was noted by [20] and validated by [21] as follows [Equation (1)]:

(1) $$\frac{I(D)}{I(G)}=\frac{C\left(\lambda_{L}\right)}{L_{a}}$$

The parameter C (λ_L_ = 514 nm), which corresponds to ~ 44 Å, represents the wavelength dependent prefactor. The wavelength dependency of C was considered by [24], who reported the following relation: C(λ_L_) ≈ C_0_ + λ_L_C_1_, where C_0_ = −12.6 nm and C_1_ = 0.033, valid for 400 nm < λ_L_ < 700 nm [24,25].

The results obtained for our samples by applying Equation (1) are shown in Table 1. Our data show that graphite is nanometric with a crystallite size ranging from an average of 138(24) nm of AhS 209b to 67(8) nm of AhS 72 and 84(14) nm of AhS A135A.

### 3.3. Geothermometry Application to Graphite in AhS Ureilite

A geothermometer for determining the maximum temperature (T_max_) of the parent body of carbonaceous matter in chondrites was developed by [18]. In their study [18], they proposed that a unique spectroscopic feature identified by studying twenty-five different samples of meteoritic insoluble organic matter (IOM) through carbon X-ray absorption near edge structure (XANES) spectroscopy provided what these authors considered a good estimate of the parent body metamorphism. Applying their approach to previously published micro-Raman data by [26], they were able to calibrate a new thermometric equation, which leads to a self-consistent organic derived temperature scale. [18] assumed that the error (2σ) associated with the use of Γ_G_ is relatively large, as ±120°C, is the uncertainty represented by the distribution of experimental points of their curve, see Equation (5) by [18]. Although the analytical uncertainty of this method is large, this geothermometer allows the determination of much higher temperatures than well-established methods used on terrestrial metamorphic graphite, which only permit the studying of samples of lower temperatures, e.g., 650 °C [27]. We also note that the temperature estimates made with this thermometer could be affected by defects induced during polishing. Thus, again, it is crucial that our data were obtained on non-polished graphite samples.

The equation of [18] is expressed in terms of Raman G-band full width at half maximum (hereafter Γ_G_) as follows [Equation (2)]:

(2) $$T_{max}\left({}^{\circ}\text{C}\right)=1594.4-20.4\Gamma_{\text{G}}-5.8\times 10^{-2}\Gamma_{\text{G}}^{2}$$

Equation (2) was applied by [19] to non-polished graphite in AhS ureilite sample #7, resulting in an average temperature of 990 ± 120 °C.

Table 2 reports the positions of graphite peaks (G-band, D-band and D^′^ band) and the relevant Γ_G_ values for all our studied samples and the T_max_ estimated temperature using Equation (2) of [19]. In order to compare our Γ_G_ data with those published by [19], we corrected our data for the instrumental peak broadening using a high-quality gemstone lithospheric diamond, following the same procedure as in [19]. These authors reported for a lithospheric diamond a Γ_G_ value equal to 3 cm^−1^; our measurement on a lithospheric diamond provides a Γ_G_ value equal to 6 cm^−1^. Therefore, in Table 2, we report both uncorrected and corrected data. The T_max_ calculations were performed using corrected data (last column in Table 2).

Our calculations indicate an average temperature of 1266 °C for AhS 209b, 1242 °C for AhS 72 and 1332 °C for AhS A135A. The standard deviations of the measurements for these three average values are 77°C, 46°C and 28 °C, respectively. However, the analytical temperature uncertainties of ± 120 (2σ) °C estimated for this experimental approach by [18] are much higher.

## 4. Discussion

Comparing our results with the previous temperature estimate on AhS ureilite #7, obtained using the same technique [19], it is evident that our temperature data are higher by at least two uncertainty intervals, i.e., ~1240–1330 °C for our samples vs. 990 °C for AhS #7 [19]. Our temperature data are within the range of peak equilibration temperatures of ureilites recorded by pyroxene geothermometry [7,28,29], whereas the AhS #7 temperature [19] is lower.

The apparent agreement between the temperatures obtained on graphite by micro-Raman spectroscopy in this work and those obtained by pyroxene geothermometry in ureilites suggests the possibility that the graphite temperatures could record the temperature of the UPB due to internal heating/differentiation. However, our Raman data not only provide a temperature estimate recorded by graphite, but at the same time they also tell us that graphite is nanometric, which strongly suggests that this graphite is the product of some transformation from an original carbon compound. In detail, the geo-thermometer by [18] is based on graphite’s G-band FWHM, which cannot be the same for recrystallized nanographite and original crystalline graphite in the ureilitic parent body. Consequently, as nanographite was reduced in size by the shock, the temperature recorded by this nanographite can be ascribed to the shock itself. For this reason, the temperatures we have estimated could represent the temperature recorded by graphite during a shock event. Graphite existing within the mantle of a planetesimal under conditions of high static pressure and temperature for millions of years, as inferred for the igneous stage of ureilite evolution [30,31], would not be expected to be nanometric in grain size, but rather to develop into much coarser crystals. Indeed, mm sized crystals of well-crystalline graphite, such as in very low-shock ureilites, are inferred to have been the primary igneous form of graphite in all ureilites [12], whereas the graphite in all shocked ureilites has been found to be internally polycrystalline and fine-grained [32]. In our samples, which have undergone a significant shock event [17], these primary graphite grains have been internally recrystallized to much smaller grain sizes, presumably during the shock process. This is also supported by the findings of [16] and [17] that this graphite is intimately associated with nanodiamonds, which were demonstrated to have plausibly formed by transformation from a pristine form of carbon (likely larger, well-crystalline graphite crystals) due to a shock event. Therefore, it seems unlikely that the temperatures recorded by nanometric graphite in our study correspond to the temperatures of pristine UPB. Although high shock pressures could also be accompanied by high-temperature regimes, our temperatures (e.g., 1200–1300 °C) are consistent with a shock event characterized by pressures as low as 15–20 GPa (determined by the AhS 72 and AhS 209b samples based on olivine mosaicism [17,33]). The evidence that high pressure could be accompanied by high-temperature regimes is well explained by [34] in their Figure 5, in which they reported the P-T Hugoniot curve for some rocks (e.g., gabbros, basalt, mare basalt, granite). Among them, there is also the Murchison carbonaceous chondrite (CC) (composed of olivine, pyroxenes and carbon phases), with a mineral association similar to that of an ureilite but with a considerably higher porosity in a larger matrix in respect to an ureilite. Using the data by [34], for our average temperatures between 1242 and 1332 °C, the returned shock pressure is between 21 and 23 GPa, respectively. However, it is known that a higher porosity and matrix of the carbonaceous chondrites [35,36] could increase the temperature during a shock event. Therefore, if we consider these rheological differences between Murchison CC and ureilites, the pressures that refer to our estimated temperatures are underestimated. However, these are still consistent with the pressures derived from olivine mosaicism, which we observed in our meteorites (≥15 GPa) [17,33].

If our interpretation is correct, however, it begs the question as to why the graphite in AhS #7 [19] records a lower temperature than our samples, when AhS #7 appears to be of the same, high-shock level as AhS 72 and AhS 209b [37]. This question would require further investigation of the grain size of graphite in AhS #7 and a detailed comparison of shock features. Indeed, a comprehensive MRS study of graphite in ureilitic samples of a wide range of shock levels, including the least-shocked, is needed to fully understand the process of the resetting of MRS graphite temperatures by shock. In addition, it could be possible to compare the temperature recorded by graphite with the temperature estimated on pyroxenes using other geo-thermometric approaches [7]. Our results clearly suggest that this would be a fruitful area for future work and could have applications in other graphite-bearing meteorites.

An alternative mechanism is that the nanometric graphite could have formed from back-transformation of diamonds after the pressure was released. Based on recent works that have focused on the thermal stability of diamonds [38–40], it is proposed that nanodiamonds start to graphitize above 800 °C; however, such a process, analyzed by high-resolution transmission electron microscopy, is characterized by the presence of an “onion graphite structure”. Such a structure was not observed by TEM in our samples, see [17]. A second scenario of diamond graphitization could refer to graphitization from a large pristine microdiamond but the temperatures recorded by the graphite of our samples were close to 1200–1300°C and this range of temperature, according to [39], is not enough to induce graphitization on a microdiamond, and for this process temperatures above 1500°C are required. The temperature obtained in this work on graphite, close to 1240–1330 °C (±120 °C), could represent the temperature related to the shock event or, following [34], it could be the post-shock temperature.

## 5. Conclusions

In this work, we investigated, by Raman spectroscopy, unpolished fragments of graphite in ureilites AhS 209b, AhS 72 and AhS A135A. AhS 209b and AhS 72 were recently studied by [16], who characterized them by X-ray diffraction and determined that these fragments consisted mainly of intimately associated nanodiamond and nanographite. Graphite in our AhS ureilite samples is nanometric with a crystallite size ranging between about 70 and 140 nm.

Our micro-Raman study on graphite provided the following results: all samples showed homogeneous values of G-band centers (between 1577 and 1585 cm^−1^) and D-band centers (between 1351 and 1357 cm^−1^); the Γ_G_ values of graphite for the G-band provided temperatures between 1242 and 1332 °C (±120 °C), which is two sigma higher than previous temperature estimates.

The mineral association of nanodiamonds and nanographite in ureilites points to the production of an impact event. Although the obtained temperature is similar to the reported igneous equilibration temperatures of ureilites [7], the observation that graphite in our sample is nanometric suggests the temperature recorded in the crystallization structure of the graphite is imprinted by the shock wave. This imprinting likely occurred during the strongest impact event it experienced during its history, which was probably the one disrupting the ureilite parent body.

## Figures and Tables

**Table 1.** Integrated intensities of the D- and G-bands, (I(D)/I(G) which is the ratio of the integrated intensities of the D and G band and the crystallite size of graphite (L_a_) of all micro-Raman Spectroscopy (MRS) acquisitions of AhS samples. For the intensity ratios, I(D)/I(G), the standard deviation is 0.08. For La, the estimated uncertainty is in the order of 20 nm.

D-Band	G-Band	I(D)/I(G)	L_a_(nm)
**AhS 209 b1**			
127900	399714	0.32	138
220935	479647	0.46	96
318675	710620	0.45	98
201393	397684	0.51	87
102706	351800	0.29	151
**AhS 209b3**			
338898	653892	0.52	84
360516	578409	0.62	70
206073	433157	0.48	92
321808	605128	0.53	83
280470	519306	0.54	81
**AhS 209b4**			
668370	1164605	0.57	77
203554	355261	0.57	77
282967	417484	0.68	65
211874	463592	0.46	96
287755	442705	0.65	68
**AhS 72**			
321884	550667	0.58	75
285483	476515	0.60	73
89546	127387	0.70	63
384023	491833	0.78	56
317754	479550	0.66	66
**AhS A135A**			
20946	37139	0.56	78
23887	35184	0.68	65
23911	44283	0.54	81
8174	18598	0.44	100
9394	21367	0.46	95

**Table 2.** Center positions for G-, D- and D′-bands and Γ_G_ (both in cm^−1^) of all the studied samples. Calculated crystallization temperature, T_max_, is reported in the last column and was obtained using Equation (2). The uncertainty (2σ) of T_max_ is ±120 °C.

G-Band Center	G-Band Γ_G_	G-Band Γ_G_ Corrected	D-Band Center	D-Band Γ_G_	D′-Band Center	D′-Band Γ_G_	T_max_ (°C)
**AhS 209B**							
b1							
1582	22	11	1356	41	1615	26	1360
1582	27	13	1354	49	1618	25	1310
1582	35	18	1352	47	1619	37	1212
1582	27	13	1355	46	1618	40	1309
1582	29	15	1355	47	1618	29	1285
b3							
1585	45	23	1354	55	1618	31	1103
1583	33	17	1354	50	1620	28	1237
1581	28	14	1355	50	1620	28	1300
1583	29	15	1355	48	1620	27	1284
1583	32	16	1355	57	1621	28	1254
b4							
1581	43	22	1355	60	1613	44	1122
1582	26	13	1355	49	1619	31	1313
1582	25	12	1354	47	1618	30	1332
1580	35	17	1353	51	1619	24	1219
1580	22	11	1353	52	1611	58	1357
**AhS 72**							
1577	33	13	1352	53	1616	37	1245
1579	40	16	1351	54	1616	31	1166
1581	29	20	1353	51	1606	68	1283
1584	32	15	1352	50	1619	33	1246
1583	30	14	1353	50	1620	29	1274
**AhS A135A**							
1582	25	13	1355	48	1619	20	1320
1583	28	14	1356	51	1618	26	1288
1582	22	10	1357	41	1620	25	1350
1580	21	12	1353	57	1614	30	1361
1582	23	11	1357	38	1620	23	1340
